# Clinical Presentation and Management of Atypical Meningioma in a Five-Year-Old Child: A Case Report

**DOI:** 10.7759/cureus.88804

**Published:** 2025-07-26

**Authors:** Mohammed Bentourqi, Mohamed Moukhlissi, Meriem Bouabid, Oussama Slimani, Ahmed BenSghier, Soufiane Berhili, Loubna Mezouar

**Affiliations:** 1 Radiation Oncology, Faculty of Medicine and Pharmacy, Mohammed First University, Oujda, MAR

**Keywords:** adjuvant radiation therapy, atypical meningioma, fronto-temporo-parietal region, pediatric meningioma, surgical excision

## Abstract

While meningiomas are the most frequent among adult primary intracranial tumors, they remain a rare condition in children, accounting for less than 5% of pediatric central nervous system tumors. These tumors have been linked to conditions like neurofibromatosis type 2 and radiation exposure, and they usually present with increased intracranial pressure and other neurological symptoms. Diagnosis relies on magnetic resonance imaging (MRI), which reveals heterogeneous masses often mistaken for other types of tumors.

Surgical resection is the first line of treatment; however, it is often complicated by anatomical characteristics and the fast growth rate of pediatric meningiomas. Adjuvant radiotherapy is commonly used in high-grade or residual meningiomas. Histopathological evaluation is essential to determine the grade and subtype of the meningioma, which in turn influences the prognosis and clinical management.

The case involves a five-year-old boy suffering from a large atypical meningioma, managed successfully by complete surgical resection followed by radiotherapy. This scenario underscores the significance of integrated therapeutic modalities that bring together surgery and radiotherapy for better long-term control over the disease in children with meningioma cases.

## Introduction

Meningiomas are the most common primary intracranial tumors, accounting for over 35% of primary cranial tumors in adults [1]. Conversely, children’s cases are rare and only represent about 5% of all central nervous system (CNS) neoplasms [2]. They are occasionally associated with neurofibromatosis type 2, but it is also known that radiation exposure is one of the major risk factors for the development of meningiomas in children [3,4].

The most common presentation in children is an increase in intracranial pressure; however, neurological deficits are relatively uncommon. Pediatric meningiomas grow very fast, and frequently, their intraventricular origin leads to early obstruction of the cerebrospinal fluid. Clinical manifestations depend on the location and size of the tumor, which can produce quite a wide array of symptoms, including headache, somnolence, seizures, impairment of visual acuity, or even extremity weakness [5].

For meningiomas, magnetic resonance imaging (MRI) is the recommended diagnostic modality; contrast-enhanced computed tomography (CT) scans should only be used in situations when MRI is contraindicated. Meningiomas can appear as heterogeneous masses on imaging, which can mimic malignant gliomas [6]. Grade I tumors are slow-growing, while Grade II and Grade III tumors are more aggressive and have a significantly higher risk of recurrence, with Grade III being particularly prone to early recurrence within five years [7].

The primary treatment for meningiomas is surgical resection. Nevertheless, in children, meningioma can infiltrate the brain and so become hard to resect [7]. Radiotherapy is used for high-grade lesions, residual lesions, and recurrent cases [8]. Among the histological subtypes, meningothelial, fibrous, and transitional meningiomas are the most common, with a similar distribution in the pediatric population [8].

## Case presentation

A five-year-old male patient presented to our unit with a six-month history of progressive headaches, complicated by vomiting over the past four weeks. He also complained of progressive weakness on the right side of his body for three months. A general examination revealed macrocephaly with an occipito-frontal circumference of 54 cm. The neurological examination showed that the patient was fully conscious and well-oriented in time and space. The motor examination revealed right-sided hemiparesis with a muscle strength of 3/5 in all myotomes. The patient is right-side dominant and has no language disorder. The cranial nerve examination was normal.

**Figure 1:**
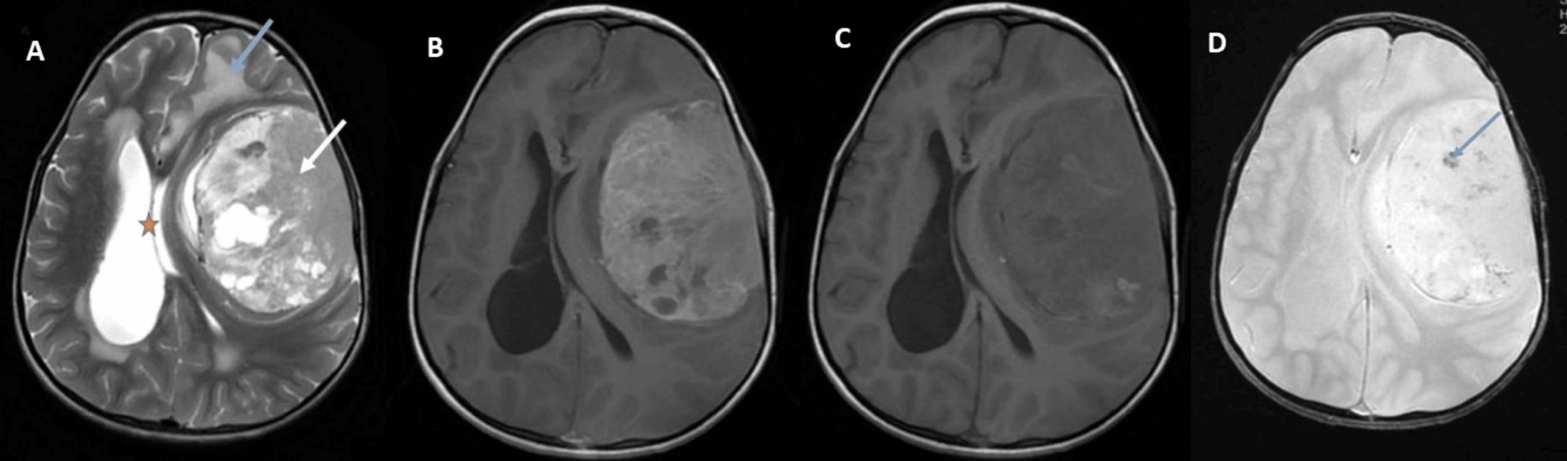
Pretherapeutic brain magnetic resonance imaging (MRI). (A) T2 axial image showing a large fronto-parieto-temporal meningioma (white arrow) in hypersignal with heterogeneous aspect with mass effect on the midline structures (red star) and peri-lesional edema (blue arrow). (B) T1 axial image showing a large fronto-parieto-temporal meningioma with iso-intense signal and heterogeneous aspect. (C) T1 axial image post contrast injection showing a large fronto-parieto-temporal meningioma with intense heterogeneous enhancement. (D) Susceptibility axial pretherapeutic image showing microcalcifications (blue arrow).

A brain MRI was performed, revealing a large extra-axial, fronto-temporo-parietal left-sided tumor process, showing heterogeneous T2 and fluid-attenuated inversion recovery (FLAIR) hyperintensity, iso-intense T1 signal, and intense heterogeneous enhancement after contrast injection, measuring 91 mm x 66 mm x 92 mm. The tumor contained some microcalcifications and was associated with significant perilesional edema, hyperostosis of the left temporo-parietal bone, and thickened meninges in the region. It exerted a significant mass effect on the midline structures, leading to severe bi-ventricular hydrocephalus with signs of transependymal resorption (Figure 1).

The surgical procedure for meningioma removal was performed under general anesthesia. The patient, positioned in the supine position, had his head slightly tilted to the right to facilitate surgical access. Rigorous preaseptic and aseptic techniques were employed before the infiltration of adrenaline-serum to minimize bleeding. A frontal arciform incision was made. Muscle disinsertion was performed. A bone flap was created to expose the dura mater, which was then coagulated and opened to access the tumor. The tumor was removed in such a way that no macroscopic residue remained, ensuring complete excision. Once the tumor was removed, the dura mater was closed, and the bone flap was replaced. A Redon drain was installed to prevent postoperative fluid accumulation. During the procedure, a blood transfusion of two units of packed red blood cells was necessary due to an incident during the operation.

The child was extubated on the second postoperative day, and a postoperative MRI confirmed that a complete macroscopic resection had been achieved (Simpson I). Histopathological analysis confirmed that the lesion was an atypical meningioma (Grade II). Over the following two weeks, the child showed progressive resolution of his right-sided hemiparesis.

**Figure 2:**
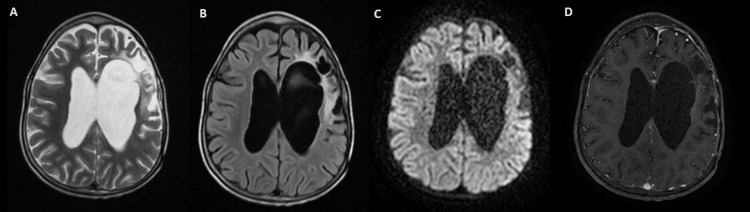
Three years post-treatment brain magnetic resonance imaging (MRI). (A) T2 axial image three years post-treatment showing hypersignal in the frontal and parietal lobes with enlarged left ventricle. (B) T1 axial image post-treatment showing frontal hypointense cavity with a surrounding hyperintense area, associated with a dilated left lateral ventricle. (C) Post-treatment diffusion-weighted imaging (DWI) axial image showing facilitated diffusion in the frontal post-treatment cavity. (D) T1 axial image post contrast injection showing no contrast enhancement in the frontal and parietal lobes.

In this case of high-grade meningioma (Grade II) in a child, the decision to administer adjuvant radiotherapy was made during a multidisciplinary meeting, given the high risk of recurrence associated with this type of tumor. The patient received irradiation to the tumor bed, with a total dose of 54 Gy, fractionated into 2 Gy per session. Radiotherapy was delivered using X-ray photon beams in a conformational technique with three oblique fields: left anterior oblique, left posterior oblique, and right lateral. The photon energies used were 6 and 18 megavolts (MV). The treatment was well tolerated, with only Grade I radiation dermatitis, which improved with symptomatic care. The initial neurological examination before radiotherapy was normal, showing a muscle strength of 5/5 across all muscle groups. The same clinical findings were observed after the completion of radiotherapy, with no neurological deficits noted. The clinical outcome, including consciousness, hearing, vision, motor function, and sensory function, was normal. The endocrine assessment, including growth hormone levels and thyroid function tests, was also normal. Three years post-treatment, the control MRI shows residual signs of left fronto-temporal surgery and a stable left fronto-temporal porencephalic cavity (49 x 18 mm) with ipsilateral lateral ventricle attraction and dilation of the lateral and third ventricles. No signs of transependymal resorption or visible obstruction are present, and the condition remains stable (Figure 2).

## Discussion

Meningiomas are frequently observed in adults, accounting for around 35% of brain tumors, but they can rarely manifest in children [4]. Among pediatric CNS tumors, meningiomas account for less than 5% of cases [8].

In children, the symptoms of meningioma are often related to seizures, intracranial hypertension syndrome, focal neurological deficits, or, more rarely, personality disorders in anterior locations. The most common manifestations include seizures, headaches, vision disturbances, vomiting, exophthalmos, and increased cranial circumference [2,9].

Pediatric meningiomas, diagnosed using imaging techniques like MRI and CT scans, share certain characteristics with adult meningiomas but exhibit notable differences [10]. On MRI, they often appear iso-hypointense on T1-weighted imaging and hyperintense on T2-weighted imaging, with frequent intense gadolinium enhancement [11]. However, a key distinction in pediatric cases is the absence of the "dural tail" or signs of dural attachment in up to 27% of cases, making neoplastic dural infiltration less consistent [10,11]. Additionally, pediatric meningiomas tend to be larger at diagnosis, with over 40% exceeding 5 cm in diameter, likely due to the child’s brain’s capacity to tolerate slow-growing masses [10]. CT scans often reveal a hyperdense mass with intense, homogeneous enhancement, alongside calcifications, edema, and hyperostosis [11]. Pediatric meningiomas are also more prone to be cystic, larger, and present without dural attachment or calcifications compared to adult cases [11].

Pediatric meningiomas are more likely to be atypical or malignant (Grade II or III) compared to adult cases, with a higher prevalence of the meningothelial subtype, followed by the transitional and atypical subtypes [2]. In the current WHO classification of CNS tumors, a meningioma is classified as Grade II atypical if it meets the following criteria: if the mitotic count is between 4 and 19 per 10 high-power fields or if there are signs of brain invasion, such as edema or necrosis, or the presence of at least three of the following histological features: increased cellularity, high nucleus-to-cytoplasm ratio, prominent nucleoli, sheet-like growth, and necrosis [12].

Ionizing radiation and neurofibromatosis type 2 are closely linked to pediatric meningiomas, with neurofibromatosis type 2 present in 21% of reported cases, often leading to multiple tumor locations. Radiation exposure, particularly after radiotherapy for other brain tumors, is another significant risk factor. However, the latency period is long, and the tumors that develop are frequently atypical or malignant [2,3,13]. Germline mutations in the NF1, SMARCE1, BAP1, and SUFU genes have also been associated with pediatric meningiomas, and the identification of clear cell meningiomas may reveal SMARCE1 gene mutations, facilitating family screening and genetic counseling [2,14].

The treatment of choice for pediatric meningiomas is total tumor resection, ideally a Simpson Grade I resection, to prevent recurrence and improve prognosis. However, this intervention is often complicated due to the large size of the tumors, their unusual location, and their close adherence to vital nerves and vessels [15]. For Grade II tumors, the five-year recurrence rate after gross total resection (GTR) increases to 50%-55%, while for Grade III, it rises to 72%-78%. When incomplete or subtotal resections are performed instead of GTR, the risk of disease progression and recurrence becomes considerably higher, further worsening the prognosis for patients with higher grades [12].

Adjuvant radiotherapy plays a critical role in the management of more aggressive meningiomas, such as atypical and anaplastic types, as well as benign Grade I tumors that cannot be fully resected. Radiotherapy following either gross total or subtotal resection has been shown to improve outcomes and stabilize lesions, as evidenced by enhanced five-year overall survival and event-free survival rates in patients with atypical and anaplastic meningiomas [16].

In summary, important prognostic factors include complete tumor resection, the histological grade, and the histological variant of the meningioma, underscoring the need for personalized and rigorous management.

## Conclusions

In conclusion, although meningiomas are rare in the pediatric population, they often present as large tumors with symptoms of increased intracranial pressure. Surgical resection remains the primary treatment, with advanced neuronavigation and microsurgical techniques improving the likelihood of successful outcomes by minimizing damage to surrounding brain tissue. Histopathological evaluation is essential in determining the tumor grade and variant, which guide management and prognosis. For high-grade meningiomas, where complete resection is challenging, adjuvant radiotherapy becomes particularly important, offering a crucial therapeutic option to reduce the risk of recurrence and improve long-term disease control. This case highlights the importance of a comprehensive approach, combining surgery and radiotherapy, in the effective treatment of pediatric meningiomas.
